# Decoding Chronicity: Oxidative Stress and Inflammation as Systems Hubs †

**DOI:** 10.3390/biomedicines13122976

**Published:** 2025-12-04

**Authors:** Dorota Formanowicz

**Affiliations:** Chair and Department of Medical Chemistry and Laboratory Medicine, Poznan University of Medical Sciences, Rokietnicka 8, 60-806 Poznań, Poland; doforman@ump.edu.pl

Chronic disorders involve complex interactions where oxidative stress, inflammation, and extracellular matrix (ECM) remodeling intersect with metabolic and immune pathways, emphasizing the need to understand these mechanisms for developing new therapies and guiding future research. A critical step toward this goal is to explore the structural and molecular frameworks where these processes converge, particularly within the vascular wall—a dynamic microenvironment where ECM proteins maintain integrity and regulate signaling. In [1], researchers demonstrated that osteopontin (OPN) and osteoprotegerin (OPG) exhibit distinct localization patterns in calcifying arteries, with OPN co-localizing with calcium deposits and OPG positioned outside these regions, suggesting complementary roles in the control of mineralization. Their quantitative analysis revealed strong correlations between OPN and elastin, as well as between OPG and collagen subtypes, highlighting that ECM remodeling is a coordinated process involving structural proteins and signaling molecules, rather than isolated events. These findings support the use of panel-based ECM phenotyping over single-marker approaches, underscoring that vascular stiffness and aging are driven by matrix changes [2].

Beyond structural remodeling, chronicity is also shaped by environmental factors that modulate inflammation. Chronic, low-level exposure to pollutants, even at concentrations considered safe, can reprogram inflammatory tone. Researchers [3] investigated the effect of silica nanoparticles (SiNPs) on airway smooth muscle cells, revealing activation of the epidermal growth factor receptor (EGFR)/proline-rich tyrosine kinase 2 (Pyk2) axis, which subsequently triggers the protein kinase C alpha (PKCα), mitogen-activated protein kinase (MAPK), and nuclear factor kappa-light-chain-enhancer of activated B cells (NF-κB), signaling cascades. This cascade leads to the upregulation of cyclooxygenase-2 (COX-2) and prostaglandin E2 (PGE2) synthesis without apparent toxicity, demonstrating how environmental nanomaterials can reprogram inflammatory and oxidative stress pathways [3]. Additionally, another study [4] provided a comprehensive overview of oxidative stress and chronic inflammation, emphasizing molecular mechanisms that counteract degenerative processes. This interplay between oxidative stress and inflammation extends to neuroinflammatory contexts, where signaling cascades drive tissue remodeling. Similarly, investigators [5] examined rhamnetin, a flavonoid with antioxidant properties, and its ability to inhibit bradykinin-induced matrix metalloproteinase-9 (MMP-9) expression in astrocytes. Mechanistically, bradykinin activates a cascade involving cellular proto-oncogene tyrosine-protein kinase (c-Src), Pyk2, EGFR, phosphoinositide 3-kinase/protein kinase B (PI3K/Akt), and c-Jun N-terminal kinase (JNK), ultimately leading to activator protein-1 (AP-1)-driven transcription of MMP-9. Rhamnetin disrupts this phosphorylation network, reducing astrocyte migration and neuroinflammatory potential.

While antioxidants can modulate inflammatory cascades, emerging evidence shows that inflammation itself is not merely suppressed but can be actively reprogrammed toward resolution. This paradigm shift introduces the concept of ‘resolution pharmacology,’ exemplified by the study [6], providing transcriptomic evidence that lidocaine, beyond its anesthetic role, promotes expression of resolution-associated genes (suppressor of cytokine signaling 1–3 (Socs1–3), dual specificity phosphatase 1 (Dusp1), tumor necrosis factor alpha-induced protein 3 (Tnfaip3)) and ECM repair markers (collagen type IV alpha 3 chain (COL4A3), collagen type VIII alpha 2 chain (COL8A2), laminin subunit beta 2 (LAMB2), and vascular endothelial growth factor A (VEGFA)) in human acute monocytic leukemia cell line (THP-1) monocytes, suggesting a transition toward an M2-like phenotype [7,8,9]. Beyond reprogramming inflammation toward resolution, chronicity may also be perpetuated by persistent infections, adding another layer of complexity.

Beyond resolution strategies, persistent infections add complexity to chronicity. While oxidative stress and inflammatory signaling dominate many chronic conditions, another layer of complexity arises from persistent infections. Viral components can sustain low-grade inflammation and alter immune homeostasis, thereby influencing the development of chronic disease phenotypes. In the study [10], authors identified Epstein–Barr virus (EBV) in the urine of 14.2% of patients with interstitial cystitis/bladder pain syndrome (IC/BPS), with viral clearance and significant reductions in interleukin 1 beta (IL-1β), interleukin 8 (IL-8), interleukin 10 (IL-10), and tumor necrosis factor alpha (TNF-α) following valacyclovir therapy. These findings highlight a viral component in chronic pain syndromes and support the use of antiviral strategies in specific phenotypes [9]. These findings emphasize that chronicity can be sustained not only by inflammatory and oxidative processes but also by persistent viral infections.

Complementary reviews advocate for multimodal care, as seen in [11], which discusses approaches for IC/BPS management, including glycosaminoglycan replenishment, botulinum toxin type A (BTX-A), and pelvic floor therapy, highlighting phenotype-oriented algorithms. Beyond these localized strategies, systems metabolic and vascular interactions reveal additional layers of chronicity. This relationship is further exacerbated as oxidative stress increases vascular stiffness and reduces nitric oxide bioavailability, thereby impairing neurovascular coupling [12].

While targeting inflammation and ECM remodeling is critical, chronicity rarely remains confined to a single organ system. Beyond localized processes, systems interactions between metabolic, cardiovascular, and neurological pathways reveal another dimension of complexity. The cardiometabolic–neurological nexus is striking: in type 2 diabetes, atrial fibrillation frequently coexists with cognitive decline, a relationship amplified by dyslipidemia. This suggests integrating vascular brain health into endocrine and cardiology care, rather than treating cognitive impairment as a late-stage consequence of these conditions. The study [13] demonstrated that patients with type 2 diabetes and atrial fibrillation exhibit more pronounced cognitive decline compared to those with diabetes alone, as assessed by Mini-Mental State Examination (MMSE) and Montreal Cognitive Assessment (MoCA) scores.

These systems interactions are mirrored along the kidney–vascular axis, where chronic kidney disease (CKD) introduces additional layers of complexity. Relationships between biomarkers and intima–media thickness (IMT) across CKD stages reveal distinct patterns that link renal dysfunction to accelerated vascular aging. In [14], the authors analyzed carotid IMT (cIMT) across CKD stages, showing that glucose concentration remains the only marker specifically associated with cardiovascular disease. At the same time, other biomarkers (urea, creatinine, phosphate, intact parathyroid hormone (iPTH), and neopterin) reflect the interplay between CKD and cardiovascular disease. Advanced oxidation protein products (AOPPs) and tissue inhibitor of matrix metalloproteinases-1 (TIMP-1) synergistically contribute to cIMT progression, reinforcing the concept of vascular aging driven by oxidative stress and ECM dysregulation. These results are consistent with the PROGression of Intima–Media Thickness (PROG-IMT) meta-analysis, which confirms that intervention-driven reductions in cIMT progression predict a lower CVD risk [15]. CKD-related vascular aging, driven by calcification and oxidative stress, further amplifies cardiovascular risk [16,17].

Collectively, these findings illustrate that chronic disorders arise from interconnected molecular networks spanning vascular, metabolic, and immune axes, underscoring the need for integrated diagnostic and therapeutic frameworks. Environmental and viral factors critically modulate immune and ECM pathways, shaping organ phenotypes and disease progression. Navigating this complexity requires systems-level methodologies, including modeling and network analysis, such as a Petri net-based approach [18,19], as well as multi-omics integration, to identify key control points and optimize interventions. To translate research into practice, three key priorities emerge: first, developing drugs that promote inflammation resolution; second, establishing precise ECM monitoring; and third, implementing diagnostic tools that integrate viral and environmental factors. Such efforts require long-term, standardized cohort studies to ensure clinical relevance and validity.

Emerging computational and pharmacological strategies further expand the therapeutic landscape. Recent advances emphasize the role of network pharmacology and integrative approaches in addressing complex chronic conditions. Mitochondrial dynamics and oxidative stress remain central to these strategies, as highlighted in [20], while ECM remodeling during fibrosis and repair introduces additional complexity [21]. Insights into free radical biology and metabolic reprogramming in inflammation are provided in [22,23], framing oxidative stress as a systems driver rather than a local phenomenon. Resolution pharmacology, including chemokine modulation, emerges as a promising avenue [24], complemented by systems perspectives on oxidative stress in chronic disease [25]. Cardiometabolic innovations underscore the need for integrated care models [26], and antioxidant-based therapeutic opportunities offer additional strategies for intervention [27]. Together, these studies outline a roadmap for multimodal interventions that bridge molecular insights with clinical translation.
